# Voluntariness or legal obligation? An ethical analysis of two instruments for fairer global access to COVID-19 vaccines

**DOI:** 10.3389/fpubh.2023.995683

**Published:** 2023-01-26

**Authors:** Katja Voit, Cristian Timmermann, Marcin Orzechowski, Florian Steger

**Affiliations:** ^1^Institute of the History, Philosophy and Ethics of Medicine, Ulm University, Ulm, Germany; ^2^Ethics of Medicine, Medical Faculty, University of Augsburg, Augsburg, Germany

**Keywords:** pandemic, COVAX, Fair Priority Model, EU regulation, ethics, equity, accountability for reasonableness, allocation

## Abstract

**Introduction:**

There is currently no binding, internationally accepted and successful approach to ensure global equitable access to healthcare during a pandemic. The aim of this ethical analysis is to bring into the discussion a legally regulated vaccine allocation as a possible strategy for equitable global access to vaccines. We focus our analysis on COVAX (COVID-19 Vaccines Global Access) and an existing EU regulation that, after adjustment, could promote global vaccine allocation.

**Methods:**

The main documents discussing the two strategies are examined with a qualitative content analysis. The ethical values reasonableness, openness and transparency, inclusiveness, responsiveness and accountability serve as categories for our ethical analysis.

**Results:**

We observed that the decision-making processes in a legal solution to expand access to vaccines would be more transparent than in COVAX initiative, would be more inclusive, especially of nation states, and the values responsiveness and accountability could be easily incorporated in the development of a new regulation.

**Discussion:**

A legal strategy that offers incentives to the pharmaceutical industry in return for global distribution of vaccines according to the Fair Priority Model is an innovative way to achieve global and equitable access to vaccines. However, in the long term, achieving the Sustainable Development Goals will require from all nations to work in solidarity to find durable solutions for global vaccine research and development. Interim solutions, such as our proposed legal strategy for equitable access to vaccines, and efforts to find long-term solutions must be advanced in parallel.

## 1. Background

The COVID-19 pandemic once again shows that the global allocation of medical goods—such as vaccines—is far from being fair and conducive to common public health goals. While rich countries have offered access to vaccines to all their citizens (1), least developed countries have not been able to guarantee adequate access even to top priority groups, such as health professionals (2, 3). Since national borders cannot contain a pandemic and other infectious diseases will emerge after COVID-19 (4), long-term strategies for global vaccine distribution are needed.

An available tool to improve access to COVID-19 vaccines in low-income countries is to issue compulsory licenses, as foreseen in the Trade-Related Aspects of Intellectual Property Rights (TRIPS) Agreement. The TRIPS Agreement of the World Trade Organization contains specified exceptions to make the protection of intellectual property compatible with responsibilities to address public health emergencies (5). Critics state that compulsory licenses are insufficient to expand access to medical goods (6, 7).

As a response to the shortcomings of compulsory licensing, the COVID-19 Vaccines Global Access Facility (COVAX) initiative has been started. COVAX, which is co-led by The Vaccine Alliance (Gavi), the Coalition for Epidemic Preparedness Innovations (CEPI), and the World Health Organization (WHO) aims to guarantee fair and equitable access to COVID-19 vaccines in each of the more than 190 voluntarily participating countries around the world, regardless of their financial resources. COVAX intended to deliver two billion doses of vaccine by the end of 2021. Initially 3% and later 20% of the population of the participating countries were to be supplied, with priority being given to healthcare workers and high-risk groups. Following, the population-based allocation criteria were to be replaced by a weighted allocation based on the countries' risk assessment (8). COVAX contributes to a more equitable global distribution of vaccines, but consistent implementation is lacking. The aim for vaccine doses delivered in 2021 was not achieved and huge inequalities remain between high-income countries and countries in the Global South (2, 9).

Neither the voluntary nature of COVAX nor the TRIPS flexibilities for public health emergencies currently lead to a fair global distribution of COVID-19 vaccines. It is therefore necessary to think about other strategies, as we are likely to face similar events in the future.

There are however legal instruments that remain underused to regulate a fair distribution of COVID-19 vaccines. The European Union (EU) has already implemented legal incentives in the past to motivate pharmaceutical companies to develop drugs for economically less profitable population groups (10, 11). Comparable regulations also exist in the United States, Japan, Australia and Singapore (12). At the EU level, improving access is possible through an ordinary legislative procedure in accordance with Article 294 Treaty on the Functioning of the EU (TFEU) (13). The enactment of an EU regulation is decided by representatives of the EU member states. The EU could create incentives for pharmaceutical companies to ensure fair global allocation through the Fair Priority Model while still serving markets in high-income countries.

The Fair Priority Model is a proposal for fair global vaccine allocation. It was developed by a group of international ethicists during the COVID-19 pandemic. Given the initial shortage of vaccine supplies, distribution follows three phases: In phase 1, the focus is on preventing deaths. A commonly used global health metric, the “Standard Expected Years of Life Lost” (SEYLL) is used here for the calculation. Phase 2 should focus on the overall economic and social impact of the pandemic. Here, the researchers propose two additional metrics in addition to SEYLL: The extent of poverty avoidance (“poverty gap”) and declines in gross national income (GNI) due to vaccine administration. In phase 3, returning to full functioning is the goal. A ranking of transmission rates is to serve as a scale here (14).

While the TRIPS waiver has already been discussed in detail in the literature (15, 16), alternative allocation approaches such as voluntariness of the COVAX model or governance through legislation have hardly been analyzed from an ethical perspective so far. We focus specifically on the decision-making processes on fair access to vaccines. The aim of this paper is to offer guidance on the different ethical values that need to be addressed while deciding over the equitable distribution of vaccines.

The following research question should be answered: To what extent do two different allocation strategies to improve access to COVID-19 vaccines meet the values of an ethical decision-making process?

**Figure 1:**
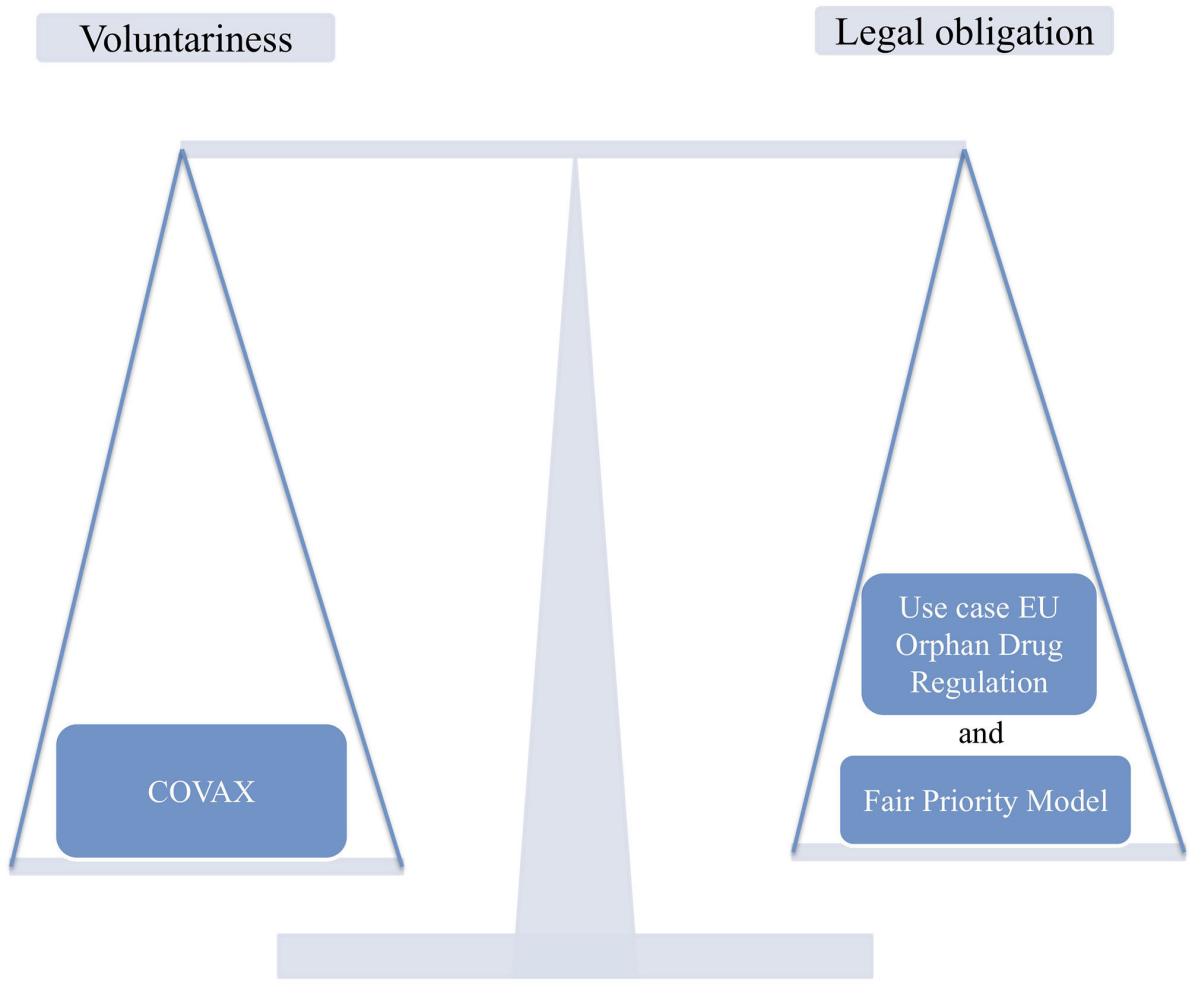
Comparison of two strategies for fairer global access to COVID-19 vaccines.

We compare a voluntary approach to global vaccine allocation, as is currently done under the COVAX program, with an existing EU regulation that, after adjustment, could promote global vaccine allocation. Therefore, we use the Regulation (EC) No. 141/2000 on orphan medicinal products (Orphan Drug Regulation), which grants incentives for pharmaceutical companies to encourage drug development and research, as a reference. In our case, incentives should be set for pharmaceutical companies that allocate vaccines globally according to the Fair Priority Model of Emanuel et al. (14) (Figure 1). Similar tools could be implemented to allocate vaccines worldwide.

To answer the research question a qualitative, structuring content analysis was performed. Our main findings are discussed in relation to global access to vaccines. The lessons learned can help to improve decision-making about fair access to vaccines or other life-saving drugs in comparable public health emergencies in the future.

## 2. Methods

**Figure 2:**
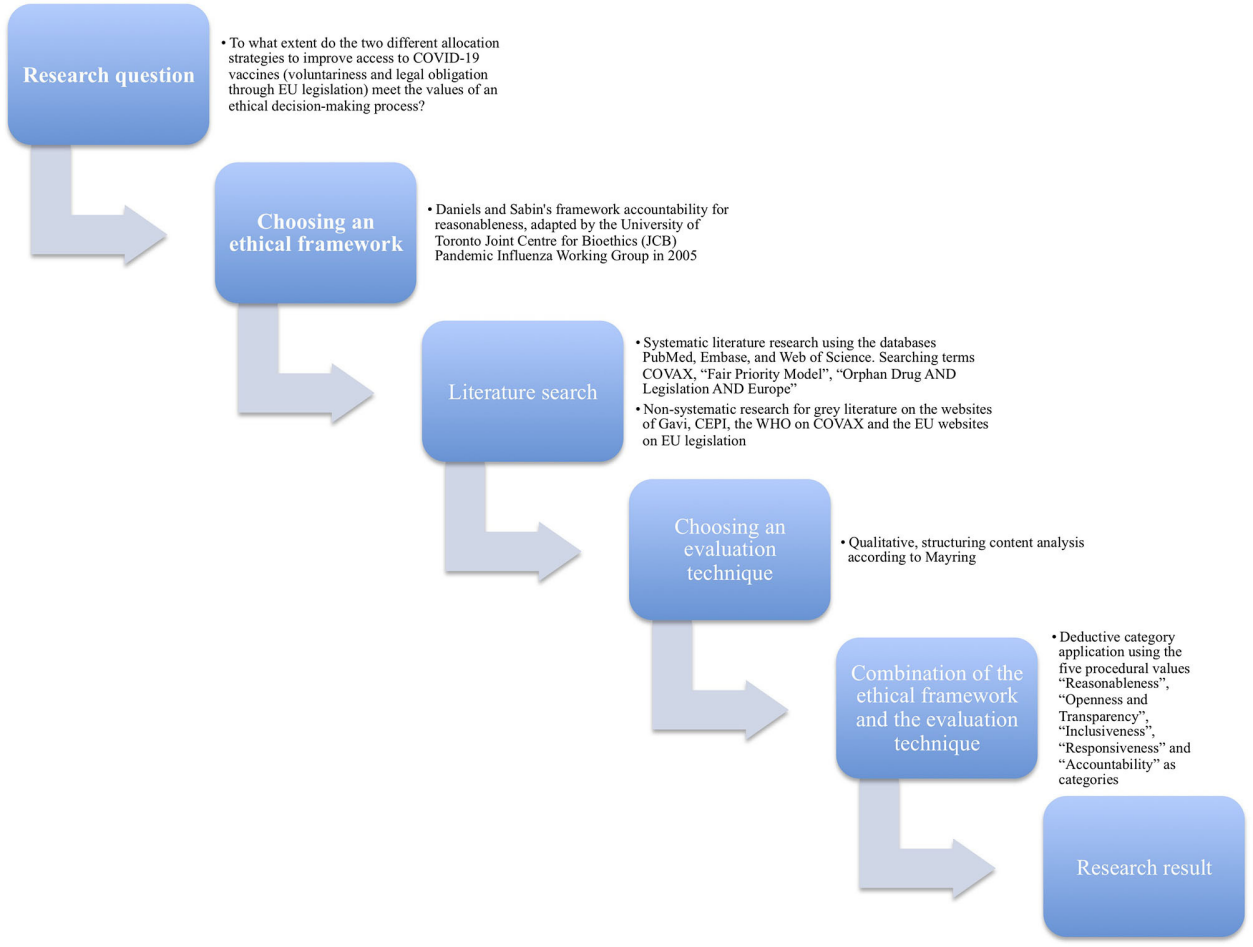
Graphical representation of the applied methodology.

This is a comparative ethical analysis of two different strategies of COVID-19 vaccine allocation. The ethical values reasonableness, openness and transparency, inclusiveness, responsiveness and accountability serve as categories for our ethical analysis. The main documents discussing the two strategies are analyzed using a qualitative content analysis (Figure 2).

### 2.1. Framework

The analysis is based on the Daniels and Sabin's framework accountability for reasonableness (17), in its adaptation by the University of Toronto Joint Center for Bioethics (JCB) Pandemic Influenza Working Group in 2005 (18). The background to this approach is that allocation decisions must be made even when there is disagreement among reasonable people about what kind of allocation strategy is ethically preferable. This framework is a proposal for a fair process for priority setting in healthcare resource allocation with far-reaching influence worldwide (19–21). By using it, it is possible to analyze the extent to which different allocation decisions regarding COVID-19 vaccines meet the ethical values of a good-decision-making process. The Canadian adaptation of the framework is based on the experiences of the Severe Acute Respiratory Syndrome (SARS) outbreak in 2002/2003 in Canada and is therefore well suited for comparable challenges in the COVID-19 pandemic. According to the JCB Working Group, for a fair decision-making process with limited resources, the following five procedural values should be met (18, 20):

- Reasonableness
- Openness and transparency
- Inclusiveness
- Responsiveness
- Accountability

We deductively analyzed these five categories to explore the extent to which the two strategies meet the values of an ethical decision-making process. The use of these original categories has been used successfully in previous research (22–24) and are considered well-established values for an ethical decision-making process. These five categories have the advantage of allowing to assess how five different values are gradually being met, permitting a full ethical analysis instead of merely constituting an ethics “tick-box” exercise. These values influence the public perception and expectations of how decisions on vaccines accessibility are done according to ethical standards.

### 2.2. Literature search

**Figure 3:**
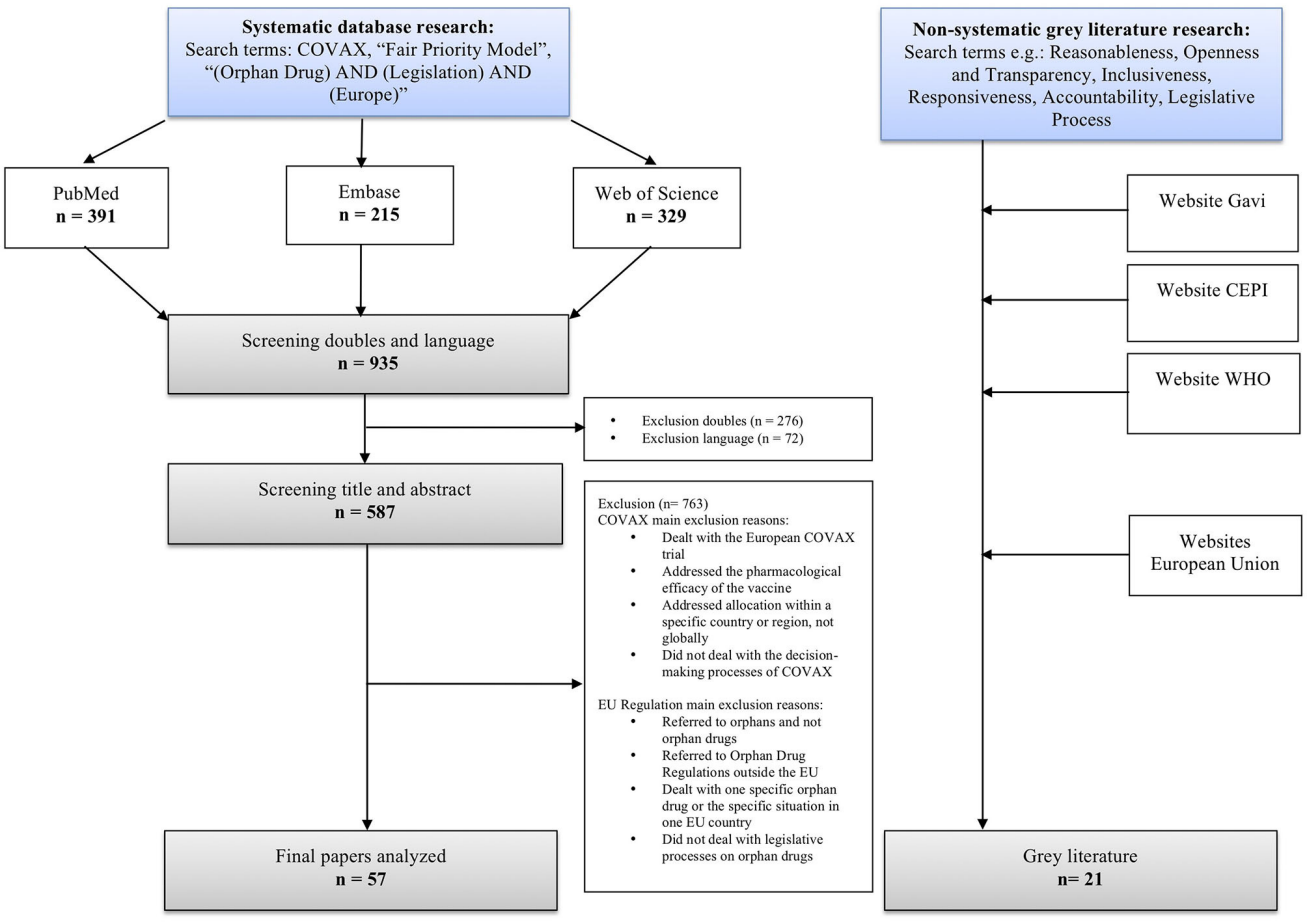
Flowchart of systematic and non-systematic literature search on May 15, 2022.

A non-standardized systematic literature search was performed on May 15, 2022, using standard settings in three of the databases: PubMed, Embase, and Web of Science. We used COVAX, “Fair Priority Model”, “Orphan Drug AND Legislation AND Europe” as searching terms. In addition, we conducted a non-systematic research for gray literature, focusing on policy reports by e.g., CEPI, Gavi, and the WHO on COVAX and the EU websites on the legislative process. The systematic literature search yielded 935 results. After removing duplicates and publications that did not meet inclusion criteria, 57 publications remained. To complement these sources, additional 21 articles from gray literature supplemented them to diversify sources (Figure 3).

### 2.3. Content analysis

A qualitative, structuring content analysis according to Mayring (25) was carried out. The five values of the adapted accountability for reasonableness approach (20) were selected as deductive categories for the ethical analysis. Associated definitions were supplemented with anchor examples from the reviewed literature and compiled in a coding guide as proposed by Mayring (25). In the event that there were delimitation problems between categories, rules were formulated in order to enable an unambiguous assignment. The coding rules were created based on the definitions of the categories and, if necessary, specified. For example, the value openness and transparency was coded with a focus on those not involved in the decision-making process and inclusiveness on those involved (Table 1). The full texts were analyzed and applicable text passages were tagged with a number that stood for one of the five ethical values. In case a statement could be classified under more than one value we opted for the most accentuated value. Disagreements in the assignment to the five values were discussed in the multi-professional team of authors consisting of experts in philosophy (C.T., F.S.), political science (M.O.), history and ethics of medicine (F.S.), and drug law and human geography (K.V.).

**Table 1:** Coding guide.

**Category/value**	**Definition adapted from Thompson et al. (20)**	**Anchor example**	**Coding rule**
1. Reasonableness	Decisions should be based on reasons that all stakeholders consider relevant to meeting health needs.	“In phase 1, we propose using Standard Expected Years of Life Lost (SEYLL) averted per dose of vaccine as the metric for premature death (...). SEYLL calculates life years lost compared to a standardized reference life table—that is, a person's life expectancy at each age as estimated on the basis of the lowest observed age-specific mortality rates anywhere in the world.” (14)	“Reasonableness” is coded for reasons that lead to the decision.
2. Openness and Transparency	The process by which decisions were made must be traceable and the reasons for decisions should be publicly available to all affected parties.	“Her main findings were that “legislative documents of the Council are not, to any significant extent, being made directly and proactively accessible to the public while the [legislative] process is ongoing”, (…), there is a certain automatic or default response of marking preparatory level legislative documents as ‘LIMITE”' [document for internal use only, not public].” (26)	“Openness and transparency” is coded as whether it is clear to people who are not directly involved in the decision-making process, who made the decision, when and why.
3. Inclusiveness	There should be opportunities to involve all affected parties in the decision-making process.	“Ahead of Gavi's board meeting on 30 July 2020, over 175 civil society organizations and individuals, including the Médecins Sans Frontières (MSF) Access Campaign, wrote an open letter to the Gavi board noting the complete absence of civil society in COVAX and demanding better representation.” (27, 28)	The involvement of those affected is coded under “inclusiveness.”
4. Responsiveness	There should be an opportunity to reconsider and revise decisions as new information emerges.	“”We all felt that now when the overall distribution is so unequal between high and lower income countries, it makes sense to really try to focus on the very lowest end countries. (…) The change marks a radical departure from the population-based approach that has been used since the first COVAX deliveries began in February.” (29)	“Responsiveness” is coded for unexpected new information.
5. Accountability	“There should be mechanisms in place to ensure that ethical decision-making is sustained throughout the crisis.” (20)	“Furthermore, though jointly initiated by multiple international organizations, the COVAX has no sufficient enforcement power to make the governments or authorities take collective binding actions, making it more like a political slogan or an initiative, rather than a feasible resolution to fight against the COVID-19 pandemic.” (30)	The mere enforcement of the decision made is coded under “accountability”.

## 3. Results

In this section, we report the results of our qualitative content analysis using five deductively formed categories (Table 2).

**Table 2:** Summary strengths and weaknesses of the decision-making process at COVAX and a legislative strategy.

**Value**	**Strengths (**+**) and Weaknesses (–)**	

	**COVAX**	**EU Regulation**
Reasonableness	**+** • Voluntary participation of over 190 countries worldwide (28, 31) • Cooperation with pharmaceutical companies (32)	**+** • A comparable regulation with incentives for pharmaceutical companies already exists (11) • Similar regulations have been enacted around the world (12) • The Fair Priority Model is based on established metrics (14) • An EU regulation has already led to improved access to medicines in the past (33–37)
	**–** • Participant commitments are not reliable (9, 38) • Pharmaceutical companies that receive grants appear to have no obligations in return (28, 39) • Nation states practice vaccination nationalism instead of global solidarity (9, 38) • Population-based allocation does not lead to equality between high-income countries and low-income countries (40, 41)	**–** • Possibly disadvantages for older people under the Fair Priority Model (42) • Theoretically available access to medicines does not succeed universally due to high prices (34, 35, 43) Wording of the regulation allows overcompensation (12, 33–35, 44)
Openness and transparency	**+** • Detailed information on the guiding principles can be found on the participating organizations' websites (45–47)	**+** • Decision-making processes laid down by law in the TFEU, the course of which is comprehensible to everyone (13, 48) • Recitals for the regulation, as well as the process and criteria for decisions are included in the regulation (11) • Largely transparent communication (13, 49)
	**–** • Selection of decision makers is unclear (50) • Agreements with pharmaceutical companies are unpublished (38, 39) • Communication is insufficient (38)	**–** • Many documents are marked for internal use only (26) • Decision-making behavior of legitimized decision-makers is not published (26)
Inclusiveness	**+**	**+** • It is possible to include all those affected (11)
	**–** • Very unbalanced involvement of stakeholders (28)	**–** • Countries outside the EU would be affected, but would not have the same decision-making power as EU countries
Responsiveness	**+** • Adjustment of initial guidelines to encourage high-income countries to participate (51)	**+** • Short-term changes in law are possible (26, 52) • Regular evaluations can be easily integrated into legislative text (11)
	**–** • Reaction to unequal vaccination progress between the countries was late (29, 39) • No preparation for possible supply bottlenecks (28, 53)	**–** • EU does not react promptly to unexpected undesirable developments
Accountability	**+**	**+** • Responsibility lies with the elected decision makers (13) • Violations of a regulation can be sanctioned (13, 54, 55)
	**–** • No superordinate, responsible institution (56) • Sanctions against voluntary participants are not enforceable	**–** • Legal text must not be formulated in such a way that it can be “exploited” and sanctions are not possible

### 3.1. Reasonableness

According to the ethical value of reasonableness, decisions should be based on reasons that can be considered intelligible by all stakeholders (20). These reasons need to be evidence-based to effectively meet health needs during a pandemic and compatible with shared values, such as human rights, solidarity and international cooperation, and non-discrimination (18, 57, 58).

Countries with delayed access to vaccines have greater potential to develop new viral variants, while coordinated vaccine sharing can reduce the risk of infection, deaths, and the financial burden of a pandemic worldwide (14, 59, 60). Because certain groups are at higher risk during a pandemic, such as the elderly, people with disabilities, the chronically ill, refugees, prison inmates, people in poor health, and healthcare professionals, these groups should have a preferential access to vaccines (21).

#### 3.1.1. COVAX and the value of reasonableness

The voluntary participation of more than 190 countries in COVAX shows that they agree in principle with the idea of a global initiative to increase access to vaccines (28, 31). Bilateral treaties and lack of compliance with COVAX principles, however, are seen as a sign that improving reputation and self-image are greater concerns than global solidarity (9, 38). Vaccine manufacturers have also announced their support for fair global vaccine distribution (61–63). However, the analyzed literature criticizes that they do not act accordingly and do not reliably support COVAX (28, 39).

It is argued that pharmaceutical companies generally lack financial incentives to develop vaccines for infectious diseases and it takes too long to develop new vaccines when relying on the market alone (64, 65). To reduce investment risk for pharmaceutical companies, COVAX, and with it public funding, has committed to purchase large quantities of vaccine upon successful development and incentivized high-risk investments in global vaccine production capacity (59, 66). Criticized are lacking mechanism to ensure sharing of knowledge and data with other companies, and impediment of vaccine donation and resale (28, 67, 68).

In practice COVAX makes a clear distinction between economically strong and weak countries (32, 66). There are not only special incentives and concessions for high-income countries, but also additional requirements for low-income countries (28, 51, 64, 69). Population-based proportional allocation as well as prioritizing healthcare workers and the elderly is criticized for giving higher shares of vaccines to countries with many health care workers and an elderly population, at the cost of countries with weak health systems and young populations (40, 41).

#### 3.1.2. Legislation and the value of reasonableness

The idea behind the orphan drugs legislation is that the pharmaceutical industry is not developing enough drugs for rare diseases under normal market conditions and therefore political intervention is required (11, 64, 70). The ten-year market exclusivity included in the regulation, which is judged to be more beneficial than patent protection, is one of several important incentives for engagement of pharmaceutical companies (33, 34, 71). The Orphan Drug Regulation is seen as positive for research and development of orphan drugs (33–37). Nevertheless, this does not result in all people within the EU having equal access to EU-approved orphan drugs; one of the reasons is the high price of many of these products (34, 35, 43). Authorities have limited negotiating power, as they do not have verifiable information on the actual development and manufacturing costs and are under pressure from patient associations and the media to approve the drugs (12).

The principle of equity and solidarity was decisive for the Orphan Drug Regulation in the EU, but also worldwide (12, 72). Patients suffering from rare diseases should have the right to the same quality of care as other patients (11). This is reflected in the Fair Priority Model: all people worldwide should have equitable access to vaccines. The strategy, which is guided by the values of benefit to the individual and limitation of harm, priority for the disadvantaged, and global equity, and aims to allocate vaccine globally using metrics such as GNI and SEYLL, was deemed effective by the model's developers (14). A critic of the Fair Priority Model points out that using SEYLL as a metric leads to a disadvantage for older people and contradicts the equal treatment of all lives (42).

The Orphan Drug Regulation contains two criteria for a designation as an orphan drug and obtain funding: Either no more than five out of ten thousand people in the EU are affected (prevalence route), or that without incentives, the drug would probably not generate sufficient profit to justify the necessary investment (return on investment route) [Article 3(1) Orphan Drug Regulation] (11). An evaluation after 20 years of Orphan Drug Regulation in Europe shows that not a single drug was brought onto the market that received support because it would otherwise not have generated sufficient profit; all benefited from the low prevalence of the diseases (44). In the opinion of many authors, the Orphan Drug Regulation leads to market failure and overcompensation, since, for example, even ibuprofen received orphan drug status and was able to benefit from privileges (12, 33–35, 44).

### 3.2. Openness and transparency

The ethical value of openness and transparency are met when decision-making processes, including arguments brought up in the deliberative processes, are disclosed. Moreover, a communication plan should be established and followed to adequately inform the participating parties about the main factors affecting the decisions (18). This increases trust toward decision-makers, decisions are more likely to be perceived as fair and it promotes solidarity (18, 21).

#### 3.2.1. COVAX in relation to openness and transparency

The analyzed literature provides detailed information on the working structure and guiding principles of the organizations involved in COVAX, as well as the rationale for global vaccine distribution (45–47, 50, 73, 74). However, the roles of the various organizations and bodies in decision-making remain unclear. It is not comprehensible for the public to identify who is responsible to whom (75). For example, there is no explanation for the selection process of committee members; unclear is also which organization or company they represent (50). The late inclusion of civil society and the participation of numerous decision-makers linked to the pharmaceutical industry (28) are not justified in detail.

The contracts with pharmaceutical companies are mostly not public, which leads to major transparency gaps (38, 39). It remains unclear according to which criteria the selection of vaccines receiving funding was made (32, 68, 69). Since the development costs are not known, it is not possible to verify whether the financial support is appropriate in relation to the incurred development costs (76). Although there is a public accountability requirement for research institutions that receive government funding, COVAX lacks transparency here (77). Furthermore, due to inclusion of vague terms in the agreements between COVAX and manufacturers, such as “statement of intent”, there are doubts about their binding nature (76). It is unclear what the consequences are for manufacturers if supply agreements are not met (68).

We found little evidence of a communication plan regarding the global distribution of vaccines (50). Minutes of the COVAX Coordination Meetings are not publicly accessible (38). Interview requests from researchers or inquiries about specific decisions at COVAX were not answered (38).

#### 3.2.2. Legislation in relation to openness and transparency

The legislative procedure for EU regulations, the legitimacy of the decision-makers and also the voting modalities are laid down by law and are thus easily comprehensible to the public [Article 294 TFEU, Articles 14 and 16 Treaty on European Union (TEU)] (13, 48). However, an evaluation in this regard revealed deficits. In particular, it was criticized that documents during the legislative process were not made sufficiently accessible to the public. It had also not been systematically recorded which positions were held by the individual member states (26). The EU citizens are therefore unable to understand the work of their elected representatives in the European Parliament (78) and influence them when necessary.

The recitals that led to the regulations are disclosed in the preamble of each EU regulation (79). In the subsequent normative part, for example, in the case of the Orphan Drug Regulation, the incentives for pharmaceutical manufacturers (Articles 7 to 9 Orphan Drug Regulation), the criteria for the designation of a drug as an orphan drug (Article 3 Orphan Drug Regulation) and who decides on this (Article 4) are regulated (11). If a new EU vaccine regulation were to be implemented, comparable standards would have to be drawn up, but in addition, the mandatory global distribution according to the Fair Priority Model would have to be made a condition. The latter is based, among other things, on the well-known parameters SEYLL and GNI (14), the calculation of which is verifiable for the public.

On the EU side, the European Commission's Directorate-General for Communication is responsible for public information (49). In addition, EU regulations are published in the Official Journal of the European Union and are accessible to everyone (Article 297(3) TFEU) (13).

### 3.3. Inclusiveness

Inclusiveness demands opportunities for all affected parties to voice their concerns and ideas in decision-making processes (20). It is intended to ensure that the decision-making process is based on ethical values shared by all those affected. Ideally, expanding participation in decision-making processes helps to balance power between different interest groups, facilitate a review by people with diverse expertise, and increase the acceptance of a decision among those who do not agree with it (20, 21).

#### 3.3.1. COVAX and the value of inclusiveness

COVAX has four groups of stakeholders: Self-funded countries, subventioned countries, vaccine manufacturers, and global health institutions (31). This leads to a mix of public and private organizations, as well as individuals without a common mission or shared values (75, 80). Key decisions are made by the “COVAX Coordination Meeting,” which includes as permanent participants the two CEOs of CEPI and Gavi, other CEPI, Gavi and WHO leaders, two representatives of the pharmaceutical industry, a member of UNICEF (United Nations International Children's Emergency Fund) and a representative of civil society (50). Of the government representatives involved in some way in COVAX's governance structure, 81% are from self-funded countries (28). The dominant role of pharmaceutical companies in the COVAX decision-making process, as well as their impact on global vaccine allocation, is frequently affirmed (28, 39, 51, 76). Civil society organizations were included in the COVAX decision-making process only after a letter of complaint was made public (28).

Although the WHO has expertise, credibility, and experience in promoting equitable access to health care (81) and acts on behalf of its members, it does not have an overarching role in COVAX (75). Nor do the states themselves have any way of influencing the contracts that COVAX signs (82). There is wide consensus on COVAX not responding sufficiently to the concerns of nation states (51, 76, 83).

#### 3.3.2. Legislation and the value of inclusiveness

Since legislation at the EU level is made jointly by the European Parliament (elected representatives of the citizens of the Union) and the European Council (representatives of the Member States at ministerial level) (Article 294 TFEU, Articles 14 and 16 TEU) (13, 48), the interests of the states are discussed in the legislative process. Further involvement of others can be defined in the legal text. For example, the Orphan Drug Regulation stipulates that a Committee for Orphan Medicinal Products (COMP) examines the extent to which medicinal products can receive orphan drug status and thus funding [Article 4(2) Orphan Drug Regulation]. Patient organizations are represented in the COMP and there is the possibility to consult experts [Article 4(3) Orphan Drug Regulation] (11).

Since the Fair Priority Model envisages global vaccine distribution (14), a solution will have to be found to include countries outside Europe in the decision-making process of an EU regulation. Comparable to COMP, a committee could be established consisting of representatives from non-EU countries. Experts from pharmaceutical companies would also need to be involved to ensure their participation and reduce opposition.

### 3.4. Responsiveness

Decision-making mechanisms can be considered to meet the value of responsiveness, when continuous review processes are implemented and decisions can be timely revised as new information emerges (18).

#### 3.4.1. COVAX and the value of responsiveness

Although it is acknowledged that global cooperation is necessary to end a pandemic, participation in COVAX was initially not attractive for many countries (51, 76). Both high- and low-income countries rather concluded bilateral agreements with pharmaceutical manufacturers (39). It required concessions on part of COVAX to get states to participate (51). For example, contrary to the original idea, signing bilateral contracts stopped being an impediment for joining COVAX (31). In addition, self-funded countries were allowed to choose a specific vaccine, although product neutrality was originally intended, and, unlike assisted countries, they were allowed to purchase vaccines for up to 50% of the population through COVAX instead of the 20% originally envisioned (51, 83).

The distribution of vaccine to all countries in proportion to population was also criticized. No distinction was made with regard to different levels of pandemic impact or existing vaccine availability. Countries with extremely low mortality rates and those who already had an adequately supply of vaccines also received their share (51, 69). Despite a shortage of available vaccine, COVAX did not initially deviate from proportional distribution. Only in mid-2021, when it became clear that < 3% of people in low-income countries received their first dose, compared with more than 60% in high-income countries, and vaccine manufacturers were prioritizing commitments to bilateral contracts before COVAX, COVAX changed its mode of distribution (29, 39). Instead of treating all countries equally and distributing vaccine proportionally to population, COVAX now focuses on countries where COVAX is the primary source—a complete departure from the original population-proportional allocation (29, 45).

Systematic preparation for possible developments is lacking: COVAX was neither prepared for rich countries buying away most of the vaccines available on the market, nor for planned deliveries not to take place (28, 53).

#### 3.4.2. Legislation and the value of responsiveness

The application of the Orphan Drug Regulation in practice revealed some unforeseen effects that need to be adjusted. It is undisputed that orphan drugs sometimes have high prices and generate very high profits (33–35, 44, 70). However, it is not possible to assess whether the prices are exaggerated because, on the one hand, data such as development costs do not have to be disclosed and, on the other hand, there is a lack of a benchmark to assess this (12, 35). In addition, the analyzed publications frequently address an unbalanced research activity with a very high concentration on oncological diseases (33, 35, 44, 72, 84). Although these aspects have been known for years, the Orphan Drug Regulation has not been adapted in this respect.

The EU needs an average of 19 months for a legislative procedure (52), in principle, however, it could adjust regulations at any time. This was proven during the COVID-19 pandemic: the parliament accepted urgent proposals within a period of 1 to 3 months, made exceptions to deadlines and, for reasons of urgency, allowed regulations to come into force on the day of their publication (26, 85).

Law can also stipulate a systematic consideration of new findings. The Orphan Drug Regulation foresees a report on its evaluation (Article 10 Orphan Drug Regulation) (11). In addition, the European Medicines Agency (EMA) regularly reports on the work of the Committee for Orphan Medicinal Products (COMP) (86). With Article 8(2) and (3), the Orphan Drug Regulation has a mechanism to ensure that it is possible to respond to changing information (11). Under certain conditions, market exclusivity can thus be withdrawn or challenged (35, 37).

### 3.5. Accountability

The value accountability is addressed when mechanism are in place to implement and enforce decisions, responsibilities are clearly defined, and decision-makers can be held accountable for their actions and omissions (18, 20, 21).

#### 3.5.1. COVAX and the value of accountability

COVAX deviates significantly in terms of accountability from the ideal. The chairs of the COVAX Coordination Meeting, the most powerful decision-making body at COVAX, are accountable only to their own organizations, CEPI and Gavi (28). The mixture of public and private organizations without legitimacy and clear responsibilities in the decision-making process jeopardizes public interests (75, 87). It is consistently criticized that there is neither a responsible higher-level organization nor an enforcement mechanism that takes effect if promises made to COVAX are not kept, so that COVAX is completely dependent on goodwill (28, 30, 38, 56, 88–90). Of the 945 million doses of vaccine pledged by high-income countries by October 2021, only 13% were delivered to COVAX (29). Calls by WHO and NGOs to wait until 10% of the world's population is vaccinated before starting with booster doses have been ineffective (59). Contradictions and violations of declarations of intent by pharmaceutical companies who announced that they would only make very small profits, would not charge excessive prices, would work for fair vaccine distribution and would supply agreed quantities cannot be sanctioned by COVAX (28, 38, 61–63, 83).

#### 3.5.2. Legislation and the value of accountability

In the case of an EU regulation, the responsibilities in the decision-making process, are clearly regulated in the TFEU, in particular in Article 294 TFEU (13). The decision-makers are legitimized by regular elections in the EU member states. However, it is questioned whether the current legislation is sufficient to effectively prevent abuse (44).

If pharmaceutical companies that receive incentives based on EU regulation violate the allocation of a vaccine according to the Fair Priority Model, this could be sanctioned. Since the primary responsibility for the application of a regulation lies with the authorities of the individual Member States (54), supplementary laws with provisions on penalties and fines may be required at national level. If an EU member state violates an EU regulation, formal infringement proceedings are initiated, which are regulated in Article 258 et seq. TFEU (13, 55).

## 4. Discussion

A legal strategy that offers incentives to the pharmaceutical industry in return for global distribution of vaccines according to the Fair Priority Model is an innovative way to achieve global and equitable access to vaccines. In our analysis of the decision-making processes of COVAX and an EU legal strategy, COVAX deviated significantly from ideal ethical decision-making processes compared to the examined legal strategy. We found significant deficits in COVAX, especially with regard to the ethical values of transparency, inclusiveness and accountability. The decision-making process in a legal regulation is more transparent than COVAX, has broader participation, especially of nation states, and the ethical values of responsiveness and accountability can easily be considered in the development of a new regulation. Therefore, we propose to use the advantages of this strategy and complement it with the positive aspects of COVAX. We continue with a discussion of the key challenges, using the five ethical values, when facilitating global access to vaccines through a legislative strategy.

Both COVAX and the Orphan Drug Regulation have strengths and weaknesses in terms of reasonableness. COVAX already showed that, in principle, there is worldwide agreement on fairer vaccine allocation. While COVAX is based on population-proportional allocation, the proposed legislative strategy relies on vaccine allocation according to the Fair Priority Model, which uses established measurements such as SEYLL and GNI. In a paper published in late 2020, scientists argued that COVAX better succeeds in uniting national and international interests (88). From today's perspective, it is obvious that COVAX has not succeeded in doing so. Instead of supporting global solidarity, nation states engaged in vaccine nationalism and “vaccine diplomacy” (38). A worldwide strategy, similar to EU regulation could be more binding when it comes to achieving the goal of a fair global distribution of vaccines according to the Fair Priority Model. Since the EU has managed to ensure equitable access to vaccines within Europe (82, 91), a similar model could be applied globally. But why should the EU take the first step? Global health is geopolitics (92). The EU claims a leadership role in the world and is committed to fair access to vaccines worldwide (93). In April 2022 the EU reconfirmed that it would also make a contribution to global solidarity in the coming pandemic phase (94). In the context of COVAX, it operated at the beginning as a mediator between international actors such as WHO, Gavi and the Gates Foundation (91). However, the EU fell behind the US and China in vaccine donations during the pandemic (38, 91, 95). If the EU took the first step in a legal strategy for global access to vaccines, it could regain diplomatic recognition and influence.

To achieve the goal of global access to vaccines, cooperation with the pharmaceutical industry is essential. The comparison with the Orphan Drug Regulation as a use case is adequate, as especially the aspect of cooperation between governments and pharmaceutical companies to improve access to medical goods can be easily transferred from orphan drugs to vaccines. From an ethical perspective, pharmaceutical companies should help optimize vaccine production, fair distribution, sustainability, and accountability in the context of COVID-19 vaccines (90). Incentives such as extended market exclusivity in a legal strategy could increase their engagement. Linking incentives to mandatory distribution of vaccines under the Fair Priority Model could simultaneously prevent pharmaceutical companies from being disproportionately favored and ensure that there is a balance between sufficient incentives and their social contribution. Undesirable overcompensation as in the Orphan Drug Regulation must be avoided. Strict attention must be paid to ensuring that vaccines are not only theoretically available worldwide, but are also financially affordable.

The challenge of disproportionately disregarding older people due to the application of SEYLL in the Fair Priority Model (42) must not be neglected. However, generally accepted criteria of equity for the distribution of limited vaccines do not exist.

The lack of openness and transparency at COVAX has led to a great loss of trust in COVAX, high-income countries and pharmaceutical companies. On the other side, although the EU legislative process is already considered as transparent, there is still room for improvement. In order to promote trust, the proposed legislative strategy should take into account the criticism of transparency mentioned in the “Activity Report: Development and Trends of the Ordinary Legislative Procedure” from 2019 (26) and initiate appropriate measures. It should ensure that fewer documents are marked “for internal use only” during the legislative process and that the public can comprehend the voting behavior of their elected representatives.

In both COVAX and a legal strategy, inclusiveness proved to be a significant ethical value. Countries worldwide would benefit from a regulation and receive a fair share of vaccine through a legislative strategy similar to the analyzed EU regulation. They could distribute it within their country according to their needs. The Fair Priority Model affects their sovereignty only in extreme cases (14). Solidarity as a justification for exclusionary decision-making processes is insufficient as a rationale. It must be ensured that the views of all recipient countries are not neglected. At the same time, this is where the greatest weakness of our regulatory approach is revealed: it prevents decolonization of global health and instead supports further dependence of low-income countries on high-income countries and so-called “vaccine apartheid” (9). Instead of promoting research and development in developing countries, as required by Goal 9 of the Sustainable Development Goals, the proposed legislative strategy, like COVAX, continues a traditional aid and charity model (38, 83, 96). Thus, this strategy can only be an interim solution on the path to the ideal: globally equitable access to vaccines achieved through global vaccine research and development (97). In the short term, COVAX was not a perfect solution, but it was a quick fix; in the medium term, a legislative strategy could bridge the gap to the long-term goal of global vaccine research and development.

A worldwide legal strategy including an allocation of vaccines according to the Fair Priority Model would meet the demands of the ethical value responsiveness well, since due to the metrics used, it is possible to react flexibly to new information or unforeseen circumstances. It is also assumed that high-income countries prefer an allocation according to the Fair Priority Model to a population-based proportional allocation used by COVAX. In the event of a major outbreak in a high-income country, the Fair Priority Model with its flexible metric SEYLL would mean preferential adjusted vaccine supply rather than suffering the disadvantages of rigid, population-based proportional allocation (98).

Furthermore, a worldwide legal strategy should include a mechanism that takes effect when it becomes apparent that access to vaccines is not possible as intended despite the regulation or unforeseen loopholes in the regulation become apparent. It seems to make sense to build standards directly into the regulation that specify a time window that must lead to an adaptation of the regulation if deficits in the regulation come to light. For example, a regular evaluation would be conceivable; in the case of the Orphan Drug Regulation, this was planned in accordance with Article 10 in 2006, 6 years after the regulation came into force (11). We think this interval is too long. Especially during a pandemic, rapid action is necessary and crucial for global access to vaccines.

This goes hand in hand with accountability. Those elected by the public to leadership roles should be aware of their responsibilities. They should ensure that everything is done to achieve fair access to vaccines and thus the best public health outcomes. This includes consistently enforcing decisions once they have been made according to ethical standards. Although sanctions are one solution to enforce goals, the example of Orphan Drug Regulation shows that they are not a general panacea for mismanagement. It is not violations by pharmaceutical companies, but unfavorable formulations in the legal text that are responsible for the identified malpractice. As a result, the original intention of the regulation, namely that patients with rare diseases have the same right to good treatment as other patients, is not achieved as well as theoretically possible (11, 33, 35, 44, 72, 84). Such misalignment would have to be avoided in any future worldwide strategy that would include comparable incentives, so that the regulation would not mainly serve economic companies but primarily equitable vaccine allocation.

## 5. Limitations

It is important to keep in mind that this research is concerned with decision-making processes on global vaccine allocation. How governments can implement these strategies in their countries, include marginalized populations, or address vaccine hesitancy was not the focus of this analysis. Moreover, a limitation of the research is the fact that it was based on the analysis of published literature. Future analysts may have access to documents which are currently not publicly available and therefore could not be assessed at this stage. This may constitute a certain bias of the results; however, the applied search strategy allowed for identification of information pertinent to the research question.

Further research is needed on how comparable legal regulations can be introduced globally. It needs to be investigated how the simultaneous development process of comparable regulations worldwide can be accelerated. Perhaps an initial coordination among the G7 countries would be a start.

## 6. Conclusions

A legal strategy offering incentives for global vaccine allocation following the Fair Priority Model of Emanuel et al. (14) is an innovative way to achieve global equitable access to vaccines. There is currently no binding, internationally accepted, and successful approach to ensure global, equitable access to healthcare during a pandemic. Current voluntary international initiatives are not sufficiently successful and have weakness concerning ethical decision-making; COVAX, for example, lacks transparency, inclusiveness and accountability.

Instead of relying on a global solution through international organizations (30, 31, 87) and patent issues (39, 51, 53), we argue for a global solution through individual nation states and associations of states that offers incentives for the pharmaceutical industry. The main advantages of the legal approach are that decisions are much more transparent, there are clear responsibilities, representatives of the nation states are legitimized by the population, and regulations can be enforced in court if necessary. Global access to vaccines would no longer depend on the unpredictable goodwill of high-income countries or commercial enterprises and private donors. In particular, we consider the possibility of imposing sanctions in our legal strategy to be advantageous if promises are not kept. This is in significant contrast to strategies currently in use, which do not have an enforcement mechanism. Patent law issues do not arise in our proposal, so that the typical problem of whether interventions in patent law are more likely to harm or benefit pharmaceutical research does not arise. In summary, the research question can be answered as follows: Both strategies need improvement in terms of adherence to ethical values in decision-making. However, COVAX is further away from meeting the central values of ethical decision-making than the examined legal strategy.

Despite the COVID-19 pandemic not being over at the time of writing (June 2022), this proposal is probably too late to implement in the current pandemic. However, our considerations are applicable to vaccine access in other pandemics and can be incorporated into pandemic preparedness guidelines for the future. Our strategy may also be of interest for other allocation issues: Vaccines are not the only medications for which global access is an issue. The same applies to many drugs, such as the new COVID-19 therapeutic Paxlovid (99).

In anticipation of the next pandemic, it is imperative that governments, involving civil society, international organizations and the pharmaceutical industry, develop global solutions that take ethical values into account. Incentives for the pharmaceutical industry to distribute vaccines globally according to the Fair Priority Model (14) could encourage them to act more fairly. However, the strategy proposed here must only be an interim solution on the way to the ideal state envisaged in the Sustainable Development Goals: equitable access to vaccines worldwide through global vaccine research and development (96). Mid-term transitional solutions, such as our legal strategy for equitable access to vaccines, and efforts to find long-term solutions need to be addressed in parallel. This requires all countries to work together in solidarity to find solutions for global vaccine research and development. As a first step, trust would have to be restored. Political will and promises as with COVAX are not sufficient for this; political action is required.

## Data availability statement

The original contributions presented in the study are included in the article/supplementary material, further inquiries can be directed to the corresponding author.

## Author contributions

Conceptualization: KV, CT, and FS. Analysis and discussion of the data: KV, CT, and MO. Writing original draft and visualization: KV. Writing review and editing: KV, CT, MO, and FS. All authors have read and agreed to the published version of the manuscript.
